# Personal Experiences of Age Discrimination in Different Life Domains: Determinants and Consequences

**DOI:** 10.1093/geroni/igab046.727

**Published:** 2021-12-17

**Authors:** Maria Clara P Paula de Couto, Jana Nikitin, Sylvie Graf, Klaus Rothermund

**Affiliations:** 1 Friedrich-Schiller University Jena, Jena, Thuringen, Germany; 2 University of Vienna, Vienna, Wien, Austria; 3 University of Bern, Bern, Bern, Switzerland

## Abstract

Age discrimination is pervasive in society which bears far-reaching consequences for individuals in terms of decreased psychological and physical health. Age discrimination can be experienced in different life-domains and perceived as a social (others’ experiences) or as a personal phenomenon (own experiences). Our first goal was to examine country- and age-related differences in personal experiences of age discrimination in distinct life domains, reported by 2,817 participants aged 40 to 90 years from the US, China, Germany, the Czech Republic, and Taiwan. As another goal, we investigated the impact of age discrimination on life satisfaction. Personal age discrimination was domain-specific, with more experiences reported in the family, work, and personality domains. Personal age discrimination increased with age and was higher in China and Taiwan. Age discrimination negatively predicted life satisfaction. This negative effect was more pronounced if age discrimination was experienced in domains with high subjective importance.

